# (2,2′-Bipyridine-κ^2^ *N*,*N*′)bis­(*N*-ethyl-*N*-methyl­dithio­carbamato-κ^2^ *S*,*S*′)zinc(II)

**DOI:** 10.1107/S1600536810002606

**Published:** 2010-01-30

**Authors:** Noorul Aisyah Abdul Ghafar, Ibrahim Baba, Bohari M. Yamin, Seik Weng Ng

**Affiliations:** a School of Chemical Sciences, Universiti Kebangbaan Malaysia, 43600 Bangi, Malaysia; b Department of Chemistry, University of Malaya, 50603 Kuala Lumpur, Malaysia

## Abstract

The complete mol­ecule of the title compound, [Zn(C_4_H_8_NS_2_)_2_(C_10_H_8_N_2_)], is generated by crystallographic twofold symmetry, with the Zn atom lying on the rotation axis; the axis also bis­ects the central C—C bond of the 2,2′-bipyridine mol­ecule. The metal atom is chelated by two *S*,*S*′-bidentate dithio­carbamate anions and the *N*,*N*′-bidentate heterocycle, resulting in a distorted *cis*-ZnN_2_S_4_ octa­hedral geometry. The methyl and ethyl groups of the anion are statistically disordered.

## Related literature

For other 2,2′-bipyridine adducts of zinc dithio­arbamates, see: Ali *et al.* (2006 ▶); Deng *et al.* (2007 ▶); Jie & Tiekink (2002 ▶); Lai & Tiekink (2004 ▶); Manohar *et al.* (1998 ▶); Thirumaran *et al.* (1999 ▶); Yin *et al.* (2004 ▶); Zemskova *et al.* (1993 ▶).
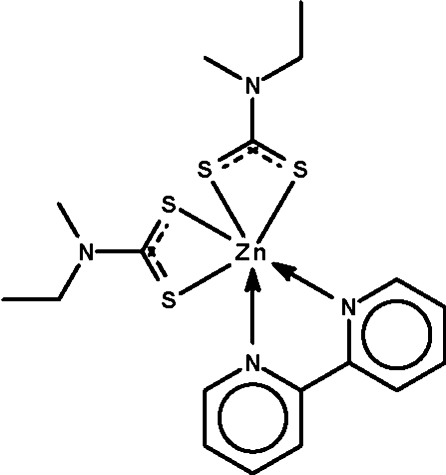


## Experimental

### 

#### Crystal data

[Zn(C_4_H_8_NS_2_)_2_(C_10_H_8_N_2_)]
*M* *_r_* = 490.02
Orthorhombic, 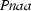

*a* = 16.9478 (7) Å
*b* = 19.3282 (8) Å
*c* = 6.6572 (3) Å
*V* = 2180.70 (16) Å^3^
*Z* = 4
Mo *K*α radiation
μ = 1.52 mm^−1^
*T* = 293 K
0.45 × 0.40 × 0.35 mm

#### Data collection

Bruker SMART APEX CCD diffractometer
Absorption correction: multi-scan (*SADABS*; Sheldrick, 1996 ▶) *T*_min_ = 0.548, *T*_max_ = 0.618
13725 measured reflections
2513 independent reflections
2252 reflections with *I* > 2σ(*I*)
*R*_int_ = 0.019

#### Refinement

*R*[*F* ^2^ > 2σ(*F* ^2^)] = 0.030
*wR*(*F* ^2^) = 0.091
*S* = 1.04
2513 reflections
136 parameters
14 restraints
H-atom parameters constrained
Δρ_max_ = 0.35 e Å^−3^
Δρ_min_ = −0.22 e Å^−3^

### 

Data collection: *SMART* (Bruker, 2000 ▶); cell refinement: *SAINT* (Bruker, 2000 ▶); data reduction: *SAINT*; program(s) used to solve structure: *SHELXS97* (Sheldrick, 2008 ▶); program(s) used to refine structure: *SHELXL97* (Sheldrick, 2008 ▶); molecular graphics: *X-SEED* (Barbour, 2001 ▶); software used to prepare material for publication: *publCIF* (Westrip, 2010 ▶).

## Supplementary Material

Crystal structure: contains datablocks global, I. DOI: 10.1107/S1600536810002606/hb5313sup1.cif

Structure factors: contains datablocks I. DOI: 10.1107/S1600536810002606/hb5313Isup2.hkl

Additional supplementary materials:  crystallographic information; 3D view; checkCIF report

## Figures and Tables

**Table:** 

Zn1—N2	2.1742 (15)
Zn1—S2	2.5259 (5)
Zn1—S1	2.5261 (6)

**Table:** 

N2—Zn1—N2^i^	75.24 (8)
S2—Zn1—S1	70.884 (17)

Symmetry code: (i) 
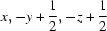
.

**Fig. 1.**
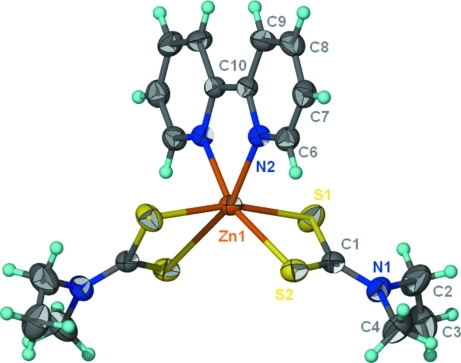
View of (I) at the 50% probability level; hydrogen atoms are drawn as spheres of arbitrary radius. The disorder is not shown.

## supplementary crystallographic information

### Experimental

Zinc chloride (10 mmol), ethylmethylamine (20 mmol), carbon disulfide (20 mmol), 2,2'-bipyridine and ammonia (10 ml) were reacted in ethanol (30 ml) at 277 K to produce a white solid. This was collected and recrystallized from ethanol to yield colourless blocks of (I).

### Refinement

Carbon-bound H-atoms were placed in calculated positions (C—H 0.93 to 0.97 Å) and were included in the refinement in the riding model approximation, with *U*(H) set to 1.2 to 1.5*U*(C). The methyl group is disordered with respect to the ethyl group. Both were refined as ethyl groups, but the methyl carbon atoms were refined with 0.5 occupancy each. The carbon-carbon distance was restrained to 1.50±0.01 Å; the anisotorpic temperature factors of the half-occupancy atoms were restrained to be nearly isotropic.

### Crystal data

**Table:**

[Zn(C_4_H_8_NS_2_)_2_(C_10_H_8_N_2_)]	*F*(000) = 1016
*M**_r_* = 490.02	*D*_x_ = 1.493 Mg m^−^^3^
Orthorhombic, *P**n**a**a*	Mo *K*α radiation, λ = 0.71073 Å
Hall symbol: -P 2ac 2bc	Cell parameters from 6268 reflections
*a* = 16.9478 (7) Å	θ = 2.4–27.5°
*b* = 19.3282 (8) Å	µ = 1.52 mm^−^^1^
*c* = 6.6572 (3) Å	*T* = 293 K
*V* = 2180.70 (16) Å^3^	Block, colorless
*Z* = 4	0.45 × 0.40 × 0.35 mm

### Data collection

**Table:**

Bruker SMART APEX CCD diffractometer	2513 independent reflections
Radiation source: fine-focus sealed tube	2252 reflections with *I* > 2σ(*I*)
graphite	*R*_int_ = 0.019
ω scans	θ_max_ = 27.5°, θ_min_ = 2.1°
Absorption correction: multi-scan (*SADABS*; Sheldrick, 1996)	*h* = −20→22
*T*_min_ = 0.548, *T*_max_ = 0.618	*k* = −25→24
13725 measured reflections	*l* = −5→8

### Refinement

**Table:**

Refinement on *F*^2^	Primary atom site location: structure-invariant direct methods
Least-squares matrix: full	Secondary atom site location: difference Fourier map
*R*[*F*^2^ > 2σ(*F*^2^)] = 0.030	Hydrogen site location: inferred from neighbouring sites
*wR*(*F*^2^) = 0.091	H-atom parameters constrained
*S* = 1.04	*w* = 1/[σ^2^(*F*_o_^2^) + (0.0554*P*)^2^ + 0.6285*P*] where *P* = (*F*_o_^2^ + 2*F*_c_^2^)/3
2513 reflections	(Δ/σ)_max_ = 0.001
136 parameters	Δρ_max_ = 0.35 e Å^−^^3^
14 restraints	Δρ_min_ = −0.22 e Å^−^^3^

### Fractional atomic coordinates and isotropic or equivalent isotropic displacement parameters (Å^2^)

**Table:**

	*x*	*y*	*z*	*U*_iso_*/*U*_eq_	Occ. (<1)
Zn1	0.292606 (17)	0.2500	0.2500	0.03998 (12)	
S1	0.27352 (4)	0.14351 (3)	0.03544 (9)	0.05648 (17)	
S2	0.19702 (3)	0.16932 (3)	0.42289 (8)	0.04706 (15)	
N1	0.16951 (11)	0.05783 (10)	0.2003 (3)	0.0545 (4)	
N2	0.39422 (8)	0.21503 (8)	0.4216 (2)	0.0379 (3)	
C1	0.20922 (11)	0.11716 (11)	0.2174 (3)	0.0424 (4)	
C2	0.18288 (18)	0.00855 (16)	0.0390 (5)	0.0884 (10)	0.50
H2A	0.2286	0.0238	−0.0367	0.106*	0.50
H2B	0.1961	−0.0357	0.0990	0.106*	0.50
C3	0.1206 (3)	−0.0020 (3)	−0.0968 (8)	0.0731 (15)	0.50
H3A	0.1326	−0.0407	−0.1819	0.110*	0.50
H3B	0.1136	0.0387	−0.1775	0.110*	0.50
H3C	0.0729	−0.0113	−0.0237	0.110*	0.50
C2'	0.18288 (18)	0.00855 (16)	0.0390 (5)	0.0884 (10)	0.50
H2'A	0.1652	−0.0364	0.0804	0.106*	0.50
H2'B	0.2382	0.0068	0.0082	0.106*	0.50
H2'C	0.1541	0.0227	−0.0781	0.106*	0.50
C4	0.11331 (16)	0.03509 (15)	0.3511 (4)	0.0725 (7)	0.50
H4A	0.1398	0.0296	0.4775	0.087*	0.50
H4B	0.0908	−0.0083	0.3109	0.087*	0.50
H4C	0.0722	0.0690	0.3644	0.087*	0.50
C4'	0.11331 (16)	0.03509 (15)	0.3511 (4)	0.0725 (7)	0.50
H4'A	0.1228	−0.0134	0.3796	0.087*	0.50
H4'B	0.1228	0.0608	0.4739	0.087*	0.50
C5'	0.0321 (4)	0.0434 (5)	0.2957 (14)	0.114 (3)	0.50
H5'A	−0.0004	0.0154	0.3816	0.171*	0.50
H5'B	0.0249	0.0293	0.1587	0.171*	0.50
H5'C	0.0173	0.0911	0.3100	0.171*	0.50
C6	0.39024 (12)	0.18074 (10)	0.5947 (3)	0.0453 (4)	
H6	0.3408	0.1703	0.6470	0.054*	
C7	0.45602 (13)	0.16000 (11)	0.6997 (3)	0.0492 (5)	
H7	0.4511	0.1362	0.8205	0.059*	
C8	0.52930 (13)	0.17520 (11)	0.6222 (3)	0.0512 (5)	
H8	0.5748	0.1617	0.6897	0.061*	
C9	0.53413 (11)	0.21061 (11)	0.4434 (3)	0.0468 (4)	
H9	0.5831	0.2212	0.3882	0.056*	
C10	0.46553 (10)	0.23031 (9)	0.3464 (3)	0.0367 (4)	

### Atomic displacement parameters (Å^2^)

**Table:**

	*U*^11^	*U*^22^	*U*^33^	*U*^12^	*U*^13^	*U*^23^
Zn1	0.03265 (18)	0.04134 (19)	0.0460 (2)	0.000	0.000	0.00225 (12)
S1	0.0583 (3)	0.0555 (3)	0.0557 (3)	−0.0150 (2)	0.0193 (3)	−0.0093 (2)
S2	0.0409 (3)	0.0566 (3)	0.0437 (3)	−0.0025 (2)	0.00315 (19)	−0.0026 (2)
N1	0.0479 (10)	0.0455 (9)	0.0701 (12)	−0.0080 (8)	0.0102 (9)	−0.0039 (8)
N2	0.0354 (7)	0.0381 (7)	0.0402 (8)	−0.0013 (6)	0.0004 (6)	0.0002 (6)
C1	0.0357 (9)	0.0422 (10)	0.0493 (10)	0.0005 (7)	0.0022 (7)	0.0024 (8)
C2	0.0759 (18)	0.0659 (16)	0.123 (3)	−0.0218 (14)	0.0324 (18)	−0.0391 (18)
C3	0.097 (4)	0.063 (3)	0.059 (3)	0.017 (3)	−0.009 (3)	−0.012 (2)
C2'	0.0759 (18)	0.0659 (16)	0.123 (3)	−0.0218 (14)	0.0324 (18)	−0.0391 (18)
C4	0.0698 (16)	0.0695 (15)	0.0783 (17)	−0.0251 (13)	0.0123 (14)	0.0122 (13)
C4'	0.0698 (16)	0.0695 (15)	0.0783 (17)	−0.0251 (13)	0.0123 (14)	0.0122 (13)
C5'	0.086 (4)	0.124 (6)	0.132 (6)	−0.010 (4)	0.025 (4)	0.026 (5)
C6	0.0455 (10)	0.0459 (10)	0.0444 (10)	−0.0039 (8)	0.0010 (8)	0.0047 (8)
C7	0.0581 (12)	0.0442 (10)	0.0453 (10)	−0.0011 (8)	−0.0068 (9)	0.0067 (9)
C8	0.0490 (11)	0.0497 (11)	0.0549 (12)	0.0086 (9)	−0.0128 (10)	0.0034 (9)
C9	0.0347 (9)	0.0524 (11)	0.0533 (11)	0.0049 (8)	−0.0018 (8)	0.0007 (9)
C10	0.0342 (8)	0.0355 (8)	0.0402 (10)	0.0007 (6)	−0.0008 (7)	−0.0024 (7)

### Geometric parameters (Å, °)

**Table:**

Zn1—N2	2.1742 (15)	C3—H3B	0.9600
Zn1—N2^i^	2.1742 (15)	C3—H3C	0.9600
Zn1—S2	2.5259 (5)	C4—H4A	0.9600
Zn1—S2^i^	2.5259 (5)	C4—H4B	0.9600
Zn1—S1	2.5261 (6)	C4—H4C	0.9600
Zn1—S1^i^	2.5261 (6)	C5'—H5'A	0.9600
S1—C1	1.707 (2)	C5'—H5'B	0.9600
S2—C1	1.712 (2)	C5'—H5'C	0.9600
N1—C1	1.334 (3)	C6—C7	1.376 (3)
N1—C4	1.452 (3)	C6—H6	0.9300
N1—C2	1.453 (3)	C7—C8	1.377 (3)
N2—C6	1.331 (2)	C7—H7	0.9300
N2—C10	1.341 (2)	C8—C9	1.375 (3)
C2—C3	1.405 (5)	C8—H8	0.9300
C2—H2A	0.9700	C9—C10	1.384 (3)
C2—H2B	0.9700	C9—H9	0.9300
C3—H3A	0.9600	C10—C10^i^	1.492 (4)
			
N2—Zn1—N2^i^	75.24 (8)	C3—C2—H2A	108.1
N2—Zn1—S2	94.40 (4)	N1—C2—H2A	108.1
N2^i^—Zn1—S2	159.93 (4)	C3—C2—H2B	108.1
N2—Zn1—S2^i^	159.93 (4)	N1—C2—H2B	108.1
N2^i^—Zn1—S2^i^	94.40 (4)	H2A—C2—H2B	107.3
S2—Zn1—S2^i^	100.22 (3)	N1—C4—H4A	109.5
N2—Zn1—S1	98.35 (4)	N1—C4—H4B	109.5
N2^i^—Zn1—S1	93.31 (4)	N1—C4—H4C	109.5
S2—Zn1—S1	70.884 (17)	H5'A—C5'—H5'B	109.5
S2^i^—Zn1—S1	99.39 (2)	H5'A—C5'—H5'C	109.5
N2—Zn1—S1^i^	93.31 (4)	H5'B—C5'—H5'C	109.5
N2^i^—Zn1—S1^i^	98.35 (4)	N2—C6—C7	122.96 (18)
S2—Zn1—S1^i^	99.39 (2)	N2—C6—H6	118.5
S2^i^—Zn1—S1^i^	70.884 (17)	C7—C6—H6	118.5
S1—Zn1—S1^i^	165.28 (3)	C8—C7—C6	118.6 (2)
C1—S1—Zn1	85.62 (7)	C8—C7—H7	120.7
C1—S2—Zn1	85.53 (7)	C6—C7—H7	120.7
C1—N1—C4	122.2 (2)	C7—C8—C9	118.97 (19)
C1—N1—C2	123.2 (2)	C7—C8—H8	120.5
C4—N1—C2	114.5 (2)	C9—C8—H8	120.5
C6—N2—C10	118.59 (16)	C8—C9—C10	119.40 (19)
C6—N2—Zn1	124.71 (12)	C8—C9—H9	120.3
C10—N2—Zn1	116.70 (12)	C10—C9—H9	120.3
N1—C1—S2	120.88 (16)	N2—C10—C9	121.49 (18)
N1—C1—S1	121.19 (16)	N2—C10—C10^i^	115.69 (10)
S2—C1—S1	117.93 (12)	C9—C10—C10^i^	122.82 (12)
C3—C2—N1	117.0 (3)		
			
N2—Zn1—S1—C1	92.98 (8)	C2—N1—C1—S2	−174.1 (2)
N2^i^—Zn1—S1—C1	168.53 (8)	C4—N1—C1—S1	−178.68 (19)
S2—Zn1—S1—C1	1.19 (7)	C2—N1—C1—S1	6.0 (3)
S2^i^—Zn1—S1—C1	−96.46 (7)	Zn1—S2—C1—N1	−178.01 (18)
S1^i^—Zn1—S1—C1	−49.05 (7)	Zn1—S2—C1—S1	1.87 (11)
N2—Zn1—S2—C1	−98.50 (8)	Zn1—S1—C1—N1	178.01 (18)
N2^i^—Zn1—S2—C1	−40.79 (14)	Zn1—S1—C1—S2	−1.87 (11)
S2^i^—Zn1—S2—C1	95.31 (7)	C1—N1—C2—C3	−114.6 (4)
S1—Zn1—S2—C1	−1.18 (7)	C4—N1—C2—C3	69.8 (4)
S1^i^—Zn1—S2—C1	167.40 (7)	C10—N2—C6—C7	−0.3 (3)
N2^i^—Zn1—N2—C6	179.12 (19)	Zn1—N2—C6—C7	−179.41 (16)
S2—Zn1—N2—C6	−18.34 (15)	N2—C6—C7—C8	−0.2 (3)
S2^i^—Zn1—N2—C6	118.46 (16)	C6—C7—C8—C9	0.2 (3)
S1—Zn1—N2—C6	−89.64 (15)	C7—C8—C9—C10	0.3 (3)
S1^i^—Zn1—N2—C6	81.35 (15)	C6—N2—C10—C9	0.7 (3)
N2^i^—Zn1—N2—C10	−0.03 (10)	Zn1—N2—C10—C9	179.93 (14)
S2—Zn1—N2—C10	162.50 (13)	C6—N2—C10—C10^i^	−179.12 (19)
S2^i^—Zn1—N2—C10	−60.7 (2)	Zn1—N2—C10—C10^i^	0.1 (3)
S1—Zn1—N2—C10	91.21 (13)	C8—C9—C10—N2	−0.7 (3)
S1^i^—Zn1—N2—C10	−97.80 (13)	C8—C9—C10—C10^i^	179.1 (2)
C4—N1—C1—S2	1.2 (3)		

Symmetry codes: (i) *x*, −*y*+1/2, −*z*+1/2.
